# *metanetwork*: A R package dedicated to handling and representing trophic metanetworks

**DOI:** 10.1002/ece3.10229

**Published:** 2023-08-15

**Authors:** Marc Ohlmann, Jimmy Garnier, Laurent Vuillon

**Affiliations:** ^1^ Laboratoire d'Écologie Alpine, LECA, CNRS Univ. Savoie Mont Blanc, Univ. Grenoble Alpes Grenoble France; ^2^ Laboratoire de Mathématiques, LAMA, CNRS Univ. Savoie Mont Blanc, Univ. Grenoble Alpes Chambéry France

**Keywords:** network layout algorithm, network visualisation, node embedding, R package, trophic metanetworks, trophic networks

## Abstract

Trophic networks describe interactions between species at a given location and time. Due to environmental changes, anthropogenic perturbations or sampling effects, trophic networks may vary in space and time. The collection of network time series or networks in different sites thus constitutes a metanetwork. We present here the R package *metanetwork*, which will ease the representation, the exploration and analysis of trophic metanetwork data sets that are increasingly available. Our main methodological advance consists in suitable layout algorithm for trophic networks, which is based on trophic levels and dimension reduction in a graph diffusion kernel. In particular, it highlights relevant features of trophic networks (trophic levels, energetic channels). In addition, we developed tools to handle, compare visually and quantitatively and aggregate those networks. Static and dynamic visualisation functions have been developed to represent large networks. We apply our package workflow to several trophic network data sets.

## Graphical abstract

We present *metanetwork* R package, dedicated to handling and representing collection for trophic networks. We implemented a layout algorithm specifically designed for trophic networks.

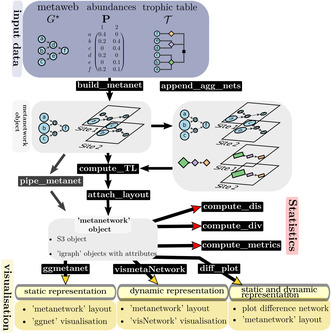

## 1 INTRODUCTION

The representation of nature was at the heart of naturalism from the *XVIII*th to the beginning of *XX*th century, mixing the need for naturalist documentation and the quest for aesthetics (Ogilvie, 2008). The representation of collections of species, in museums or in situ through plates fed the picture book of the emerging ecology. This has rooted the representation of a community as a collection of species, without considering biotic interactions. Interestingly, the plates of invertebrates by Haeckel (e.g. marine invertebrates, Haeckel, 1904) highlight the importance of geometry in representing those organisms. The emergence of community in ecology during the early *XX*th century introduces interactions between species in the representation of an ecological community (Elton, 1927). The foundations of network ecology are established. Since then, trophic interaction networks have been recognised as controlling dynamics and functioning of communities and they have been used for managing biodiversity (Polis & Winemiller, 2013; Thompson et al., 2012). Adequately representing networks is then crucial for researchers as well as for decision‐makers (Pocock et al., 2016).

The main issue in trophic network representation is still on providing a meaningful network layout related to ecological features, such as trophic levels or energetic channels (e.g. Elton, 1927; Van Leeuwen et al., 2015). Trophic networks are usually high dimensional with complex structure, while network layout is only a two‐dimensional node embedding. Although network visualisation tools are now widely available (e.g. Bastian et al., 2009; Csardi & Nepusz, 2006; Pawluczuk & Iskrzyński, 2022; Perrone et al., 2020), current network layout methods highlighting hierarchical structure of trophic networks remain scarce (but see Hudson et al., 2013). They mainly rely on force‐directed algorithms, as Fruchterman and Reingold (1991) that is based on vertex repulsion or Kamada and Kawai (1989) and Gansner et al. (2004) that consists in spring embedding. None of them incorporate ecological processes. As a result, their outcomes on trophic networks are hard to interpret since these algorithms do not model ecological processes. Node layout algorithms specifically designed for trophic networks are still lacking.

Representing networks properly is an even more important issue as they are now sampled in space and time (CaraDonna et al., 2017; Dunne, 2006) as biogeography classically represents species in space (Lomolino et al., 2017; Von Humboldt & Bonpland, 1805). Empirical evidence supports plasticity and stochasticity of interactions and would encourage sampling of trophic interactions through space and time (CaraDonna et al., 2017; de Aguiar et al., 2019; Poisot et al., 2015). However, sampling interactions in multiple sites is challenging since it requires joint observations of species. It is especially problematic when it involves organisms from different kingdoms and various body sizes (Jordano, 2016). Sampling taxa is far easier than sampling interactions, using naturalist knowledge (Moser et al., 2005), camera traps (Steenweg et al., 2017) or environmental DNA (Bohmann et al., 2014). A convenient case to study networks in space is then to build a potential network at the regional scale, the metaweb, using expert knowledge or machine learning methods to complete interaction databases (Strydom et al., 2021). Once the metaweb is built, local networks are deduced using sampled abundances. Such an approach have been used for various organisms, from terrestrial vertebrates (Braga et al., 2019; Galiana et al., 2014) to marine or freshwater communities (Blackman et al., 2022; Kéfi et al., 2015; Kortsch et al., 2019) or soil communities (Bauer et al., 2022). While losing interaction plasticity and stochasticity, local networks nevertheless have distinct structures due to sampling effect.

Hereafter, a collection of networks in space or time is called a metanetwork, as a collection of communities is called metacommunity. For simplicity, we refer to the potential interaction network as the ‘metaweb’. While trophic network databases are becoming increasingly available (Poelen et al., 2014), tools to handle and represent them remain scarce. The present paper describes and implements a new layout algorithm built for trophic networks, using trophic levels and a diffusion‐based algorithm. This contribution also describes several additional methods to handle, represent and analyse trophic metanetworks at different resolutions as suggested in the literature (Guimarães, 2020; Thompson & Townsend, 2000). All the described methods are implemented in the R package, *metanetwork*, that eases manipulation and representation of trophic metanetworks. *metanetwork* is available on CRAN while several vignettes on several open data sets are accessible online at https://marcohlmann.github.io/metanetwork/.

We first describe inputs and methods to build and handle *metanetwork* objects. We then focus on the proposed ‘TL‐tse’ and ‘group‐TL‐tsne’ layout algorithms and the visualisation methods wrapped in *metanetwork*. We also illustrate the use of the package on several datasets of various dimensions, including marine, soil and vertebrate trophic networks.

## 2 PACKAGE WORKFLOW

### 2.1 Package installation and documentation

The latest stable version is available on CRAN and can be installed using:

install.packages("metanetwork").

Complete documentation along with several vignettes describing the examples of our paper is available here: https://marcohlmann.github.io/metanetwork/.

### 2.2 Defining and handling metanetworks

#### 2.2.1 Inputs of the ‘metanetwork’ object

To build a potential metanetwork (hereafter metanetwork), we need a metaweb, G*, that is a directed and connected network including focal species and known potential trophic interactions in the study region. We can also include a community matrix **P**, indicating species relative abundances, and a trophic table T, indicating species belonging to broader taxonomic or functional groups. Local networks are then induced subnetworks of G* by local communities (with abundances).

**FIGURE 1:**
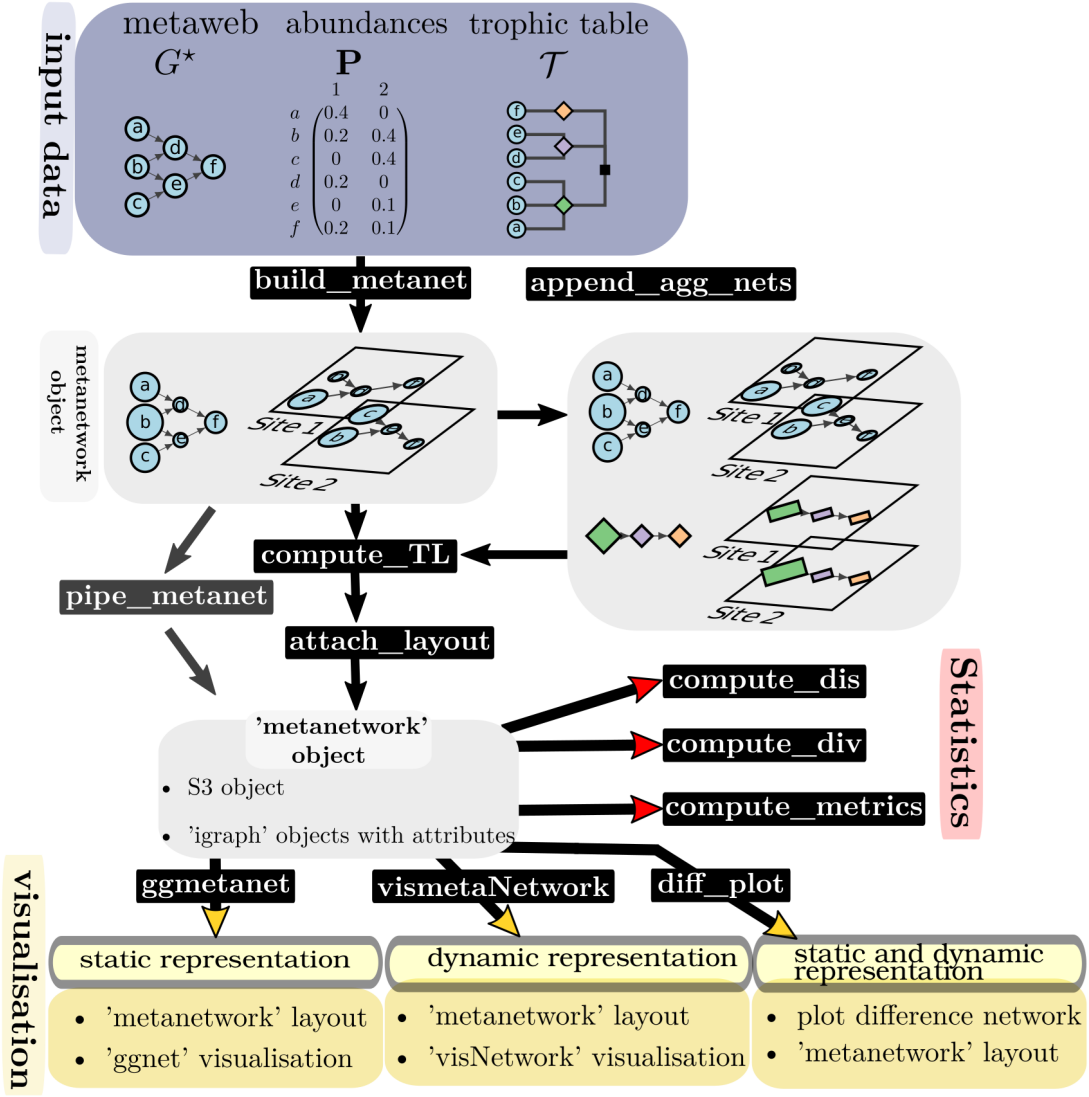
Sketch representation of the use of the R package *metanetwork* from input data to output visualisation. It highlights the main functionalities of the package to handle and represent metanetworks.

Our package encodes a metanetwork through a R S3 object of class ‘metanetwork’. The function build_metanet builds a ‘metanetwork’ object from the triplet $\left(G^{⋆},P,T\right)$ and computes local networks. The metaweb G* must be of class ‘igraph’, ‘matrix’ or ‘data.frame’. The matrix **P** and the table T can be NULL contrary to G*. In this case, the metanetwork will be a single network. Although the metaweb needs to be connected, local networks can be disconnected, which may occur due to sampling effects. Figure 1 provides a sketch representation of the package functionalities, and Table 1 describes the main functions and their associated ecological questions.

**TABLE 1:** Main functions of the *metanetwork* package and the corresponding addressed ecological questions.

Functions	Description	Ecological questions
build_metanet	Build a ‘metanetwork’ S3 object Compute local networks	What is the structure of the local networks?
append_agg_nets	Append aggregated networks to the current metanetwork using the trophic table T	What is the structure of the aggregated networks? How does it compare through aggregation levels?
plot_trophicTable	Represent aggregation levels given by the trophic table T	What are the possible aggregation levels in the metanetwork?
compute_TL	Compute trophic levels using Laplacian matrix	What are the trophic levels in the metaweb How do trophic levels vary among local networks?
attach_layout	Compute and attach ‘TL‐tsne’ or ‘group‐TL‐tsne’ layout to the current metanetwork	How are the nodes distributed along the energetic channels at a given trophic level?
ggmetanet	Static visualisation of the metaweb and the local networks using ‘ggnet’ with ‘TL‐tsne’ layout	What are the main energetic channels of the current network?
vismetaNetwork	Dynamic visualisation of metaweb and local networks using ‘visNetwork’ with ‘TL‐tsne’ layout	What are the main energetic channels of the current network?
diffplot	Compute the difference between two networks Show a static or dynamic visualisation of the different network	What are the differences between the local networks?

Local networks constitute a list of ‘igraph’ objects with relative abundances, edge weights and network names stored as node, edge and graph attributes.

#### 2.2.2 Append aggregated networks

In order to investigate trophic networks at different aggregation levels (e.g. broader taxonomic groups, functional groups or output of node clustering algorithms) as suggested in Thompson and Townsend (2000), Ohlmann et al. (2019) and Guimarães (2020), our package can compute aggregated networks using the trophic table T that describes aggregation levels (Figure 1). The nodes of the aggregated network are broader taxonomic groups given by the trophic table whereas edges are aggregated based on the structure of the original network. Considering aggregated networks is particularly welcome since nodes and edges composition can vary at the original resolution but be stable in the aggregated network. More formally, given a network *G* with *n* nodes, we can create *Q* groups from the original set of *n* nodes (Q<n) using T. We denote $\left(C_{1},\ldots ,C_{Q}\right)$ the focal groups or aggregated nodes. Their relative abundances ${\left({\tilde{p}}_{q}\right)}_{1\leq q\leq n}$ and interaction probabilities ${\left({\tilde{\pi }}_{\mathrm{ql}}\right)}_{1\leq q,l\leq n}$ are computed according to Ohlmann et al. (2019) as follows

(1) $${\tilde{p}}_{q}=\sum_{k\in C_{q}}p_{k}\;\text{and}\;{\tilde{\pi }}_{\mathrm{ql}}=\frac{\sum_{k\in C_{q},k^{\prime }\in C_{l}}\pi_{kk^{\prime }}p_{k}p_{k}^{\prime }}{\sum_{k\in C_{q}}p_{k}\sum_{k^{\prime }\in C_{l}}p_{k}^{\prime }}$$

where $\pi_{kk^{\prime }}$ is the link probability between nodes *k* in group *C*_*q*_ and nodes $k^{\prime }$ in group $C_{l}$, and $p_{k}$ and $p_{k^{\prime }}$ are their respective relative abundances.

The method append_agg_nets computes the abundances and the link probabilities at any aggregation levels provided by the trophic table T. It then appends aggregated networks with node and edge attributes to the current ‘metanetwork’ object (Figure 1).

### 2.3 Representing and analysing metanetworks

We developed and implemented a new node layout algorithm specifically for trophic networks that we called ‘TL‐tsne’, in reference to Trophic Levels and t‐sne dimension reduction algorithm. Our ‘TL‐tsne’ layout consists in a two‐dimensional node embedding algorithm. It uses the trophic levels as the *x*‐axis coordinates of the nodes in the two‐dimensional space. The coordinates on the *y*‐axis are computed using the diffusion kernel of the network (Kondor & Lafferty, 2002), which informs us on similarity between nodes according to a diffusion process, combined with a modified version of the ‘t‐sne’ algorithm, which allows reducing dimension (Van der Maaten & Hinton, 2008).

Moreover, we implemented functions to visualise and compare local networks but also metrics and indices to carry on quantitative analysis.

#### 2.3.1 Trophic levels computation

Trophic levels have been introduced to quantify the position in the hierarchy of resource acquisition (Lindeman, 1942). Despite various methods available to compute trophic levels (Hudson et al., 2013; Levine, 1980), we use the recent framework of MacKay et al. (2020), who define trophic level using the Laplacian matrix of the network because it embeds many useful properties of the network.

Let *G* be a directed network, we note **A** its adjacency matrix and **D** its degree diagonal matrix. The Laplacian matrix of the symmetrised version of *G* is defined by:

(2) $$L=D-A-t\left(A\right)$$

where $t\left(A\right)$ is the transpose of the adjacency matrix **A**. We note $v=\text{indegree}\left(G\right)-\text{outdegree}\left(G\right)$ the imbalance vector. Then, the vector of the trophic levels, **x**, is the solution of the linear system:

(3) Lx=v

For a connected network, the solution **x** is unique up to a translation. Thus, we always fix its minimal entry to 0 (corresponding to basal species) and get the trophic level of all the other ones (more details in Appendix S1). In our package, we first compute the trophic levels from the metaweb G* because this graph is connected, thus we can fix the minimal trophic level to 0 and provide a trophic level for all other species. Since local networks might be disconnected due, for instance, to sampling effects, we compute the trophic levels in each connected component of the local network, and we fix the minimal trophic level in each component to its trophic level in the metaweb graph (see Appendix S1 for more details).

The method compute_TL computes trophic levels and store them as node attributes of the networks belonging to the current ‘metanetwork’ object. These trophic levels are the *x*‐axis coordinates of our node layout.

#### 2.3.2 Diffusion graph kernel and ‘TL‐tsne’ layout algorithm

The core of our new layout method consists in the use of a diffusion graph kernel. Graph kernels consist in similarity matrices between nodes of a network based on its structural characteristics. Diffusion graph kernel computes similarity between nodes based on a diffusion process, capturing so path structure in the network (Kondor & Lafferty, 2002; Smola & Kondor, 2003). It is particularly suitable for our new network layout since it allows to cluster together nodes that are involved in similar paths. From the network *G*, we define the diffusion graph kernel **K**

(4) $$K=\exp\left(-\beta \;L\right)=\sum_{k\geq 0}\frac{{\left(-\beta \;L\right)}^{k}}{k!}$$

where **L** is the Laplacian matrix of *G* and *β* is the diffusion parameter, a scalar and strictly positive parameter. The latest part of the equation corresponds to the power series expansion of the matrix exponential, with *k* going from 0 to infinity. In our package, the diffusion kernel is computed through its eigenvalues (see Appendix S1). In the context of trophic networks, the diffusion process described by **K**, might represent diffusion of organic matter through the network. By doing so, nodes involved in the same paths (whatever their lengths) will have a high similarity. Increasing the diffusion parameter *β* will increase similarity values between nodes involved in the same paths while decreasing similarities between nodes involved in different paths. In order to compute the *y*‐axis coordinate of the nodes in our layout of the network *G*, we need to reduce the information provided by the diffusion kernel **K** (that is of dimension the node number of *G*). We use a dimension reduction algorithm adapted from the t‐sne algorithm (Van der Maaten & Hinton, 2008), which provides a low‐dimensional embedding of high‐dimensional data while preserving neighbourhood. The t‐sne method relies on an iterative algorithm, which minimises the Kullbach–Leibler divergence between similarity matrices in the high‐ and low‐dimensional space.

We use the diffusion kernel **K** to measure the similarity in the high‐dimensional space (that is the set of neighbours in our network, which is fixed). We use the same low‐dimensional similarity as in Van der Maaten and Hinton (2008). The *x*‐axis coordinate is already fixed here to the trophic levels, while the second coordinate is chosen such that the Kullbach‐Leibler divergence between the two similarity matrices is minimal. Importantly, the minimisation procedure accounts for trophic levels. We named ‘TL‐tsne’ the proposed network layout algorithm (see Algo. S1 in Appendix S1). We also provide a method to evaluate the quality of the computed layout and to select *β* value using a modified version of Moran index (De Jong et al., 1984, see Appendix S1).

The method attach_layout computes ‘TL‐tsne’ layout and store it as node attribute of the focal network.

#### 2.3.3 Visualisation

Besides proposing a new layout method, *metanetwork* package allows incorporating these layouts in the two recent R packages dedicated to network visualisation: ‘ggnet’ and ‘visNetwork’. The ‘ggnet’ package represents networks as ‘ggplot’ objects (Schloerke et al., 2018; Wickham & Wickham, 2007). Our function ggmetanet provides a static representation of the network using ‘TL‐tsne’ layout combined with ‘ggnet’ visualisation and additional features (legend, node abundances and edge weights). The ‘visNetwork’ package represents the network in an interactive way using vis.js JavaScript library (Almende et al., 2019). Our function vismetaNetwork provides ‘TL‐tsne’ layout and wraps ‘visNetwork’ dynamic visualisation with additional features (JavaScript events linked to the nodes, legend, node abundances and edge weights).

**FIGURE 2:**
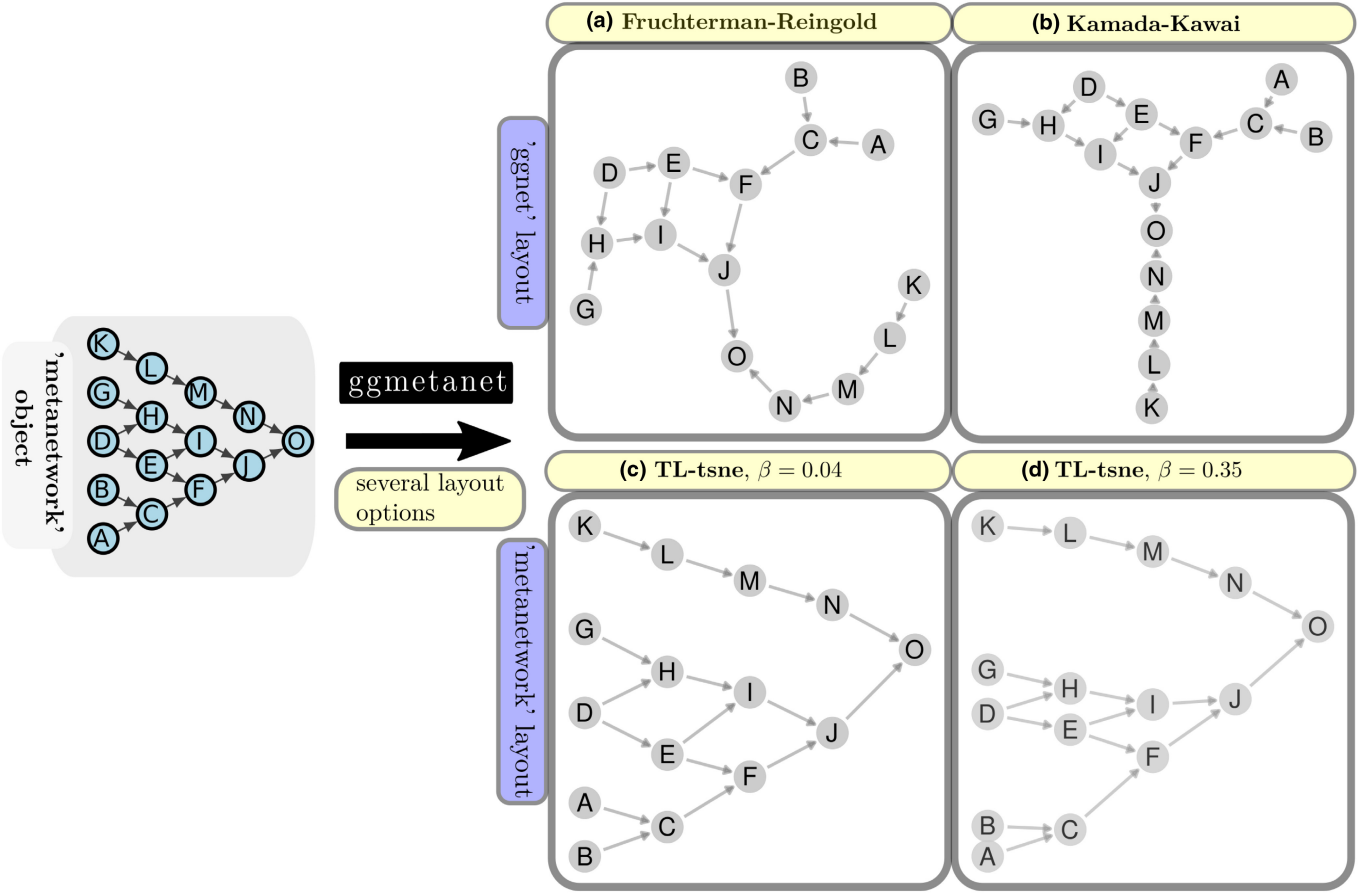
Network layout methods implemented in ‘metanetwork’ with ggmetanet visualisation function. The pyramid network example, that represents an idealised trophic network, is represented with (a) Fruchterman‐Reingold (force‐based layout), (b) Kamada‐Kawai (force‐based layout) and ‘TL‐tsne’ layout for (c) β=0.04 and (d) β=0.35.

We illustrate our layout and static visualisation functionalities on a simple directed pyramid network in Figure 2. Such network could represent an idealised trophic network with few species and a highly hierarchical structure. We represent this with the ggmetanet function, using three different layouts: Fruchterman‐Reingold, Kamada‐kawai (force‐based layouts already implemented in ggnet) and our ‘TL‐tsne’ layout with two different *β* values. Force‐based layouts (Figure 2a,b) do not capture the hierarchical structure of the network contrary to the ‘TL‐tsne’ layout (Figure 2c,d). Increasing the *β* parameter tends to gather the nodes with similar trophic levels that are involved in similar paths.

#### 2.3.4 Representing the difference between networks

In order to ease local network comparisons, *metanetwork* implements a function diff_plot that highlights differences and similarities between two network. More precisely, let $G_{1}$ and $G_{2}$ be two local networks (with vertex sets $V_{1}$ and $V_{2}$), we note $G_{\text{diff}}$ the difference network between $G_{1}$ and $G_{2}$, whose vertex set is $V_{\text{diff}}=V_{1}\cup V_{2}$. It is the induced subgraph of the metaweb, $G^{⋆}$, by $V_{\text{diff}}$. We assign then node abundances and edge weights to $G_{\text{diff}}$. Node abundance of the difference network consists in the difference between node abundances of $G_{1}$ and $G_{2}$, as edge weights. We use a colour code to distinguish nodes that are present in both networks with different abundances from nodes that are absent in one of the networks. A colour code in the visualisations indicates the sign of the node abundance difference and the edge weight difference between networks (see Figure 4 the following Section 3.1).

#### 2.3.5 Representing large networks with ‘group‐TL‐tsne’ layout

In order to represent networks with a large node number (typically larger than >100), we propose a variation of ‘TL‐tsne’ layout that uses information from trophicTable. This specific layout method, called ‘group‐TL‐tsne’ uses the ‘TL‐tsne’ layout at a desired aggregation level and combines it with ‘igraph’ layout_with_graphopt layout. We first compute the coordinates at the desired group resolution using ‘TL‐tsne’ algorithm. We then compute, in each group, the coordinates of the nodes using ‘igraph’ layout centred at the coordinate of the group. A configuration object allows playing on group diameters. The attach_layout method computes ‘group‐TL‐tsne’ layout and store it as node attribute. Computing ‘group‐TL‐tsne’ layout is more computationally efficient since it computes ‘TL‐tsne’ layout on the aggregated network (that is much smaller) only.

#### 2.3.6 Computing network metrics, diversities and dissimilarities

In order quantitatively assess network structure and compare local networks at the different resolutions, our package implements functions to compute network metrics (compute_metrics), network diversity (compute_div) and pairwise dissimilarity (compute_dis) indices (see Figure 1). compute_metrics computes mean and max trophic levels of the metaweb and local networks (using the output of compute_TL()). This function also computes mean shortest path length (using ‘igraph’ mean_distance() function). The package also implements the function compute_div that computes network diversity indices based on Hill numbers developed in Ohlmann et al. (2019). This indices can be computed both on nodes (relying then on node abundances) and edges (relying then on edge weight and node abundances), at the different network resolutions. From these indices, network pairwise dissimilarity indices are then derived (both on nodes and edges). The function compute_dis allows computing network pairwise dissimilarities at the different network resolutions.

## 3 CASE STUDIES

In this section, we apply *metanetwork* functions to three real‐world metawebs, which correspond to different ecosystems with various organisms. In the main text, we use the static representation of the networks using ggmetanet while we provide interactive visualisations using vismetanetwork online at https://shiny.osug.fr/app/ecological‐networks.

### 3.1 Angola coastal network

**FIGURE 3:**
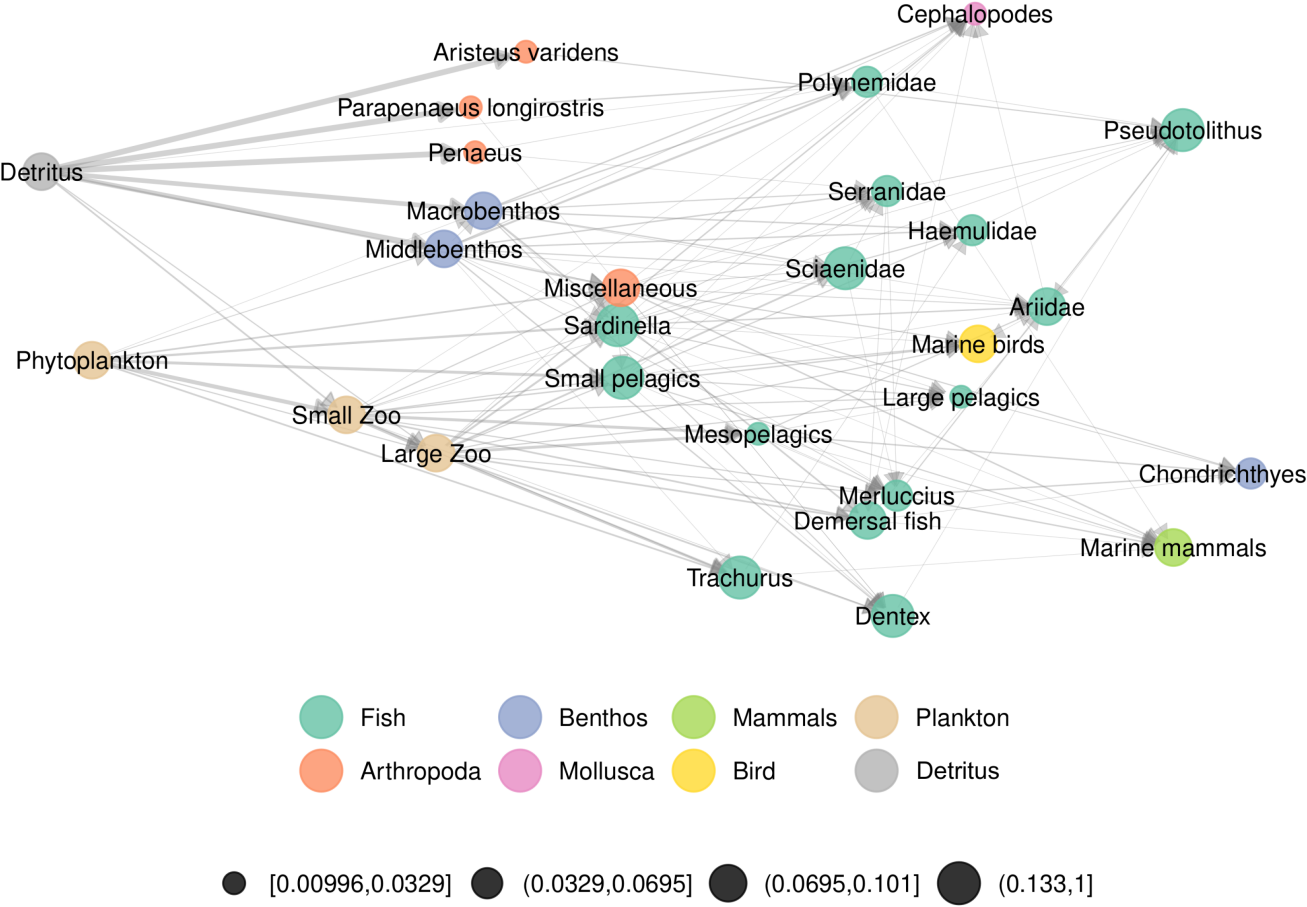
Angola coastal trophic network, which contains 28 nodes and 127 interactions. Nodes are coloured according to taxonomic groups and edges are weighted according to a diet study. We use the ‘TL‐tsne’ layout with β=0.02 and the static visualisation function ggmetanet.

**FIGURE 4:**
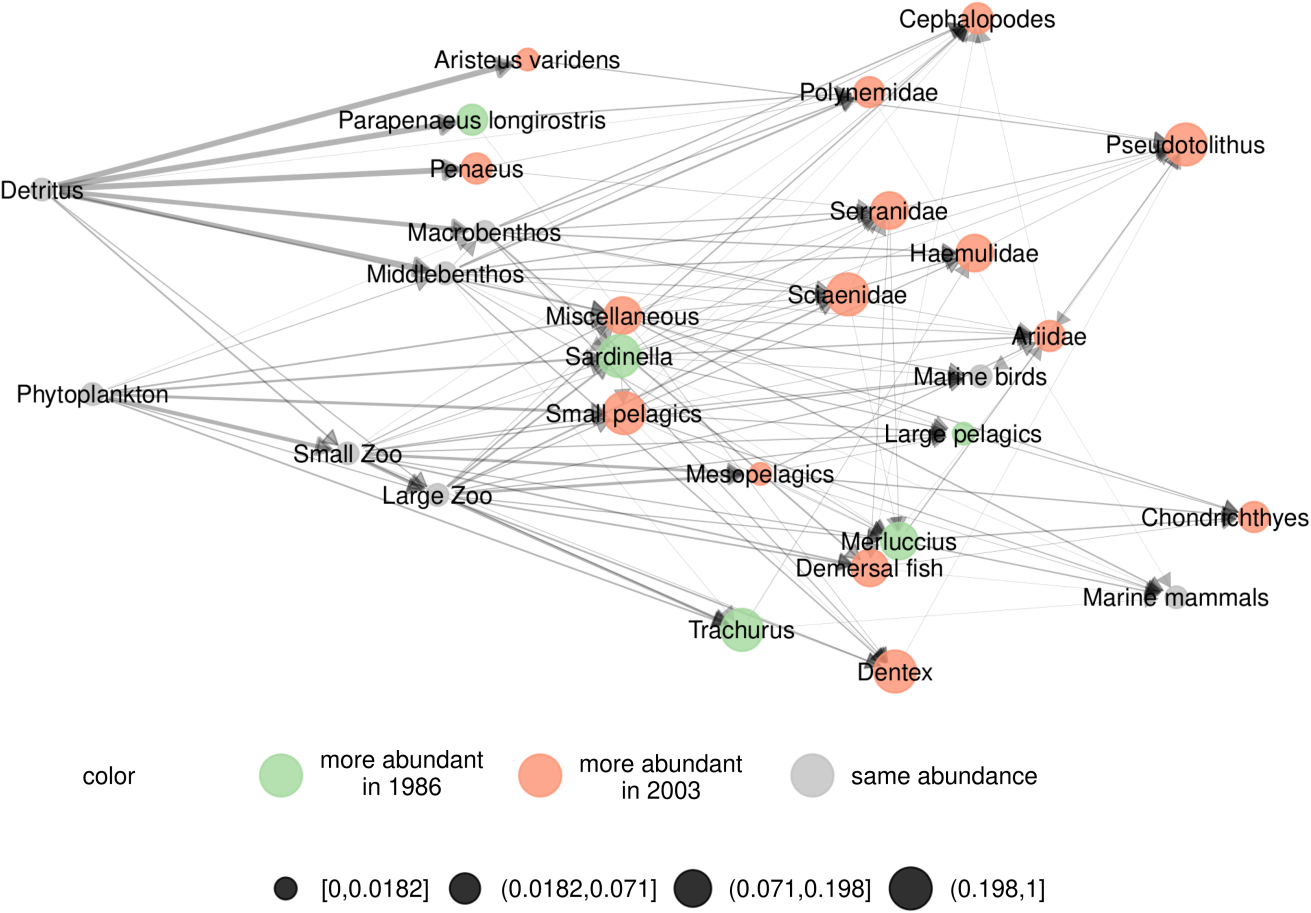
Difference network between the Angola network from 1986 and from 2003. Differences in node abundances are given by differences in estimated biomasses at the two time steps. We use the diff_plot function with the computed ‘TL‐tsne’ metaweb layout (see layout_metaweb option) to visualise the difference network.

We first look at a dataset from Angola, which has been extracted from Web of Life (http://www.web‐of‐life.es). It consists in a coastal trophic metaweb of 28 nodes (species or groups) and 127 interactions sampled along Angola's coastline (Angelini & Vaz‐Velho, 2011). The study aimed at estimating impact of Angola's fishery on the coastal trophic network by quantifying biomass using times series from multiple sources (see Angelini & Vaz‐Velho, 2011 for more details). Available abundance data consists of two time steps: 1986 and 2003. Interactions are weighted according to the relative frequency of prey species in the diet of each predator species. We represented the metaweb using ggmetanet with ‘TL‐tsne’ layout (β=0.02) in Figure 3. The metaweb has two basal nodes, ‘Phytoplankton’ and ‘Detritus’, leading to a primary producer and detritus channel that mix up higher in the network. We included the Angola dataset as an example in the package (meta_angola object), with abundances built from biomasses in 1986 and 2003. We also represented the difference network between the two dates using the diff_plot function with the ‘TL‐tsne’ layout (β=0.05) in Figure 4. We also computed a profile of extended Moran index along beta values to select optimal *β* (see Figures S2 and S3).

### 3.2 Norway soil trophic network

**FIGURE 5:**
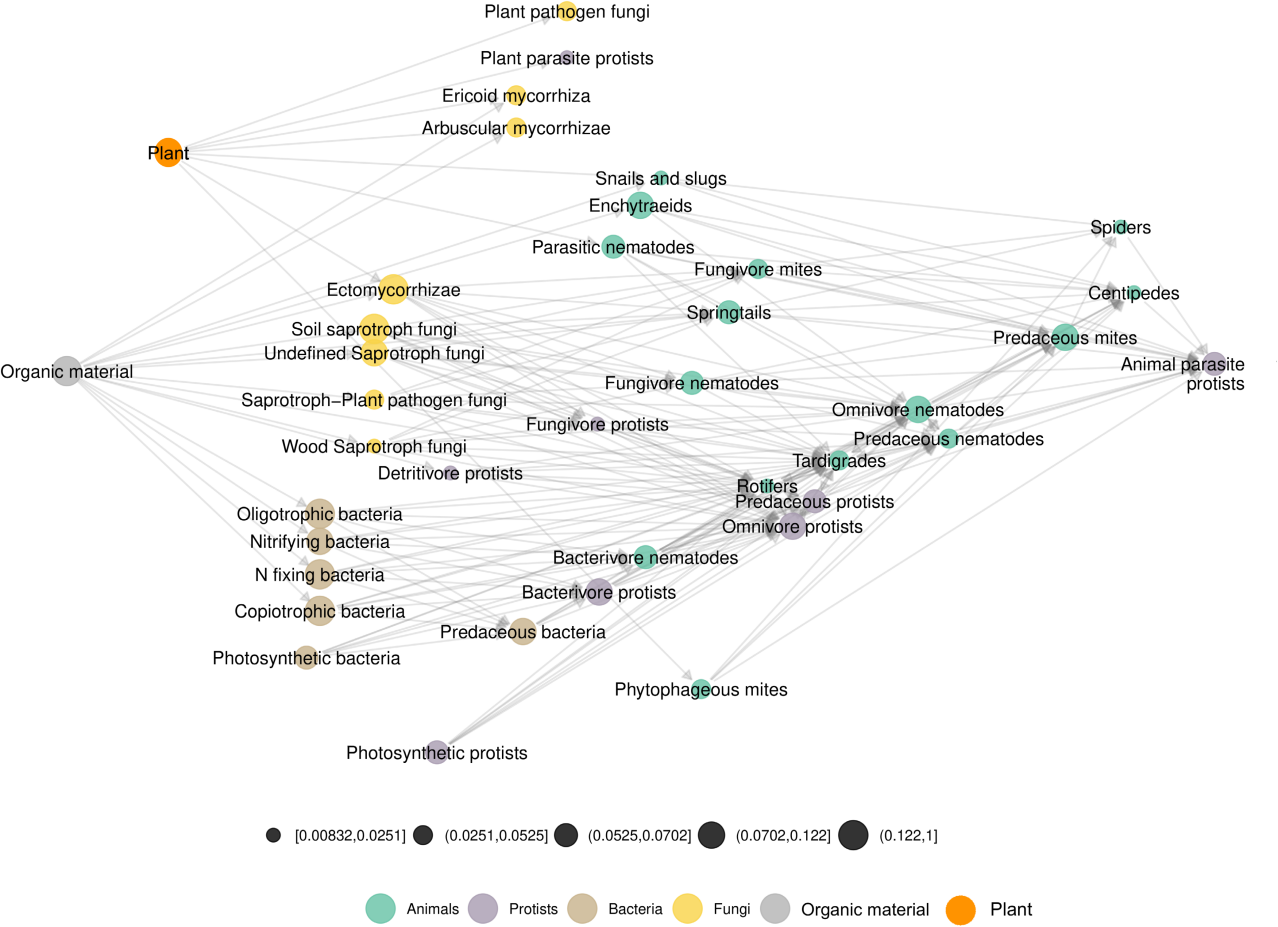
Norway soil trophic network, with 40 nodes and 204 edges. Nodes are coloured according to taxonomic groups and have relative abundances built from environmental DNA data. It is represented using ‘TL‐tsne’ layout (β=0.006) and ggmetanet visualisation.

Norway soil trophic network dataset was extracted from Calderón‐Sanou et al. (2021). It consists in a soil expert knowledge metaweb and environmental DNA data sampled in the Varanger region in Northeastern Norway. The metaweb has 40 groups and 204 interactions with several available aggregation levels (trophic group, trophic class and kingdom). The groups have relative abundances given by their mean abundances in environmental DNA samples. The Figure 5 shows the metaweb at the group level using the ‘TL‐tsne’ layout with the diffusion parameter β=0.006 (Figure 5). The metaweb has two basal resources: plant and organic material. They have the lowest *x*‐axis values in the ‘TL‐tsne’ layout. The channel starting from plants corresponds to the green energy channel while the channel starting from organic materials is the brown channel (Moore et al., 2004; Mougi, 2020; Polis & Strong, 1996). Importantly, we observe from our network representation that bacterial and fungal paths are separated in the brown channel. It means that they are linked to separated paths higher up in the network (e.g. bacterivore and fungivore groups). Calderón‐Sanou et al. (2021) documents the impact of a disturbance (moth outbreaks) on soil diversity. We provide the difference network between pre‐ and post‐disturbance (Figure S4). It highlights a shift from Ectomycorrhizae and Ericoid mycorrhizae towards Arbuscular mycorrhizae and also an increase in soil predator abundances.

### 3.3 Metaweb of European tetrapods

**FIGURE 6:**
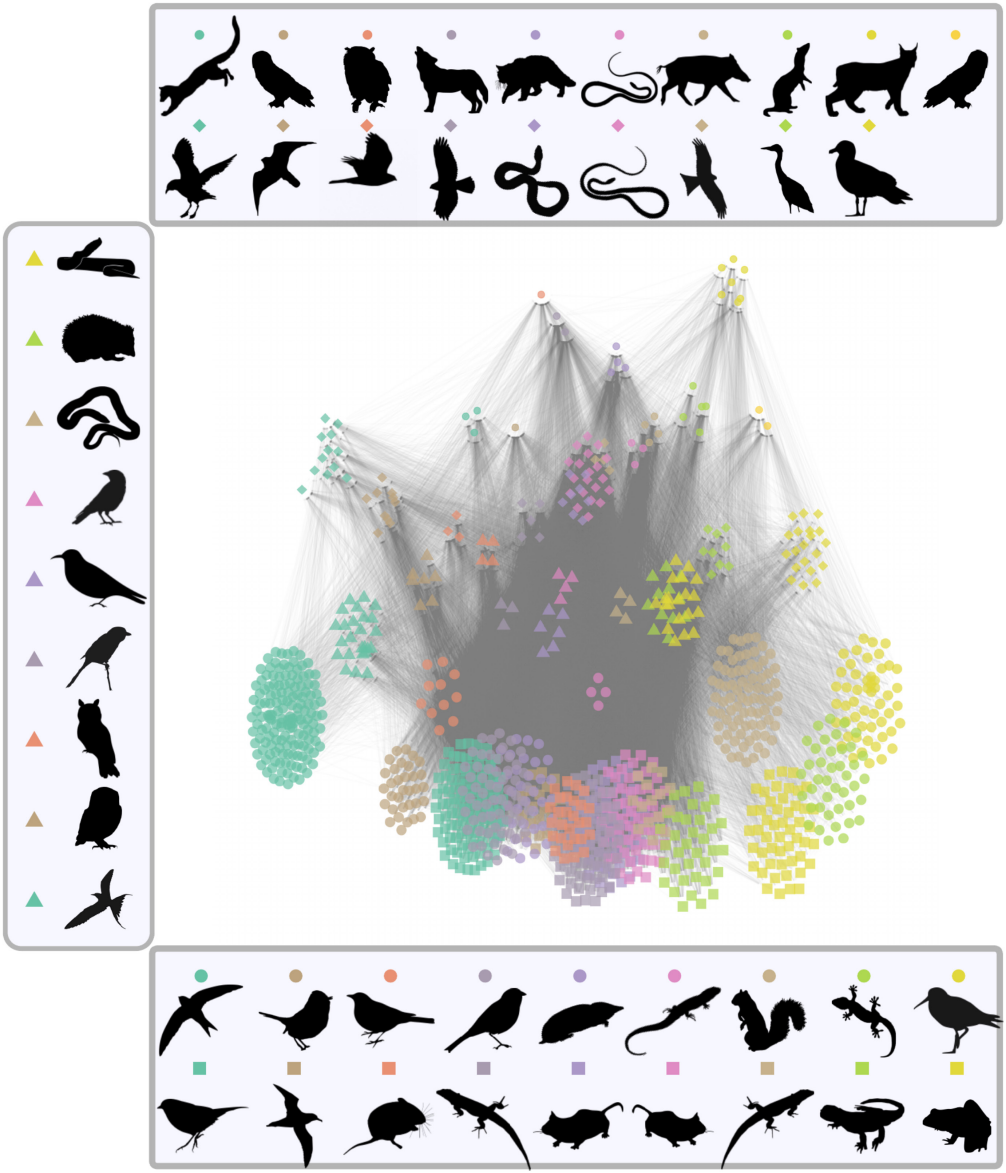
Metaweb of European tetrapods, with 1101 species (mammals, breeding birds, reptiles and amphibians) and 48,963 interactions. Nodes have colours and shapes corresponding to estimated Stochastic Block Model groups. It is represented using ‘group‐TL‐tsne’ layout, built from group layout (‘TL‐tsne’ with β=0.005) and ggmetanet visualisation. In this representation, the *y*‐axis is the trophic level. The legend is constructed by taking the silhouette of a representative of each group on http://phylopic.org/. See Table S1 for credits.

The metaweb of European tetrapods was extracted from Maiorano et al. (2020) and O'Connor et al. (2020). It consists of an expert knowledge metaweb of all tetrapods occurring in Europe (mammals, breeding birds, reptiles and amphibians) with potential interactions. This network has 1101 species and 48,963 interactions. O'Connor et al. (2020) computed trophic groups using the Stochastic Block Model (SBM) that clusters nodes with similar connectivity patterns (Daudin et al., 2008). We represented the metaweb using ‘TL‐tsne’ layout ($\beta =3\times 10^{-6}$) while flipping x and y coordinates (see flip_coords option). We mapped the 46 SBM groups using a combination of colours and shapes (see Figure S5). To get a more ordered representation, we used the ‘group‐TL‐tsne’ layout, that uses ‘TL‐tsne’ layout at a SBM group resolution (Figure 6, Figure S6). Interestingly, some SBM groups are overlaying in the ‘group‐TL‐tsne’ layout. For basal species, the group containing many rodents of genus *Microtus* (purple squares) is mixed with the group containing many rodents of genus *Spermophilus* (pink squares). Higher up in the network, the group containing predator snakes of genera *Hierophis* and *Montivipera* (pink diamonds) is overlaying with group containing snakes of genera *Vipera* and *Hemorrhois* (purple diamonds).

## 4 DISCUSSION

We have presented *metanetwork*, a R package dedicated to handling and representing trophic metanetworks. These metanetworks are built from a metaweb, an abundance table and a possible information table on nodes. Potential local networks are then deduced from the metaweb and local abundances. While loosing local plasticity of interactions, such an approach generates distinct local networks due to sampling effect. Recent studies aimed at unravelling the structure of local networks for different types of communities (Bauer et al., 2022; Kéfi et al., 2015; Kortsch et al., 2019).

The purpose of *metanetwork* R package is to provide representation tools for trophic networks and metanetworks. Representing networks consists in choosing an appropriate node layout algorithm and a suitable visualisation technique (Pocock et al., 2016). If visualisation techniques, wrapped in ‘metanetwork’, were widely available, a network layout algorithm specifically designed for trophic networks was sorely lacking. We developed the ‘TL‐tsne’ network layout algorithm, which constitutes the main methodological development of the present paper. This new layout combines the computation of trophic levels, using the Laplacian matrix on the *x*‐axis, with a non‐linear dimension reduction in the graph diffusion kernel on the *y*‐axis. Besides representing two different features, it allows reading the network along fixed axes. Our diffusion kernel method not only relies on edges, which corresponds to paths of length 1, but also on paths of arbitrary long length. As a result, our layout is less sensitive to the deletion of an edge or, more generally, to the mistakes in edge specification compared to force‐based layouts that are very sensitive, as pointed out in Pocock et al. (2016). Moreover, paths of arbitrary length do have ecological interpretations in terms of energetic channels in the network. Notice that the proposed ‘TL‐tsne’ layout uses diffusion kernel on an undirected version of the considered network on the *y*‐axis knowing the *x*‐axis that takes into account directionality of the network since an imbalance term is present in Equation (3). The present method is then only designed for directed networks. Diffusion maps achieve a similar goal for embedding of points in space relying however on an undirected graph built from spatial coordinates (Coifman et al., 2005). We also notice the proximity of our method with node embedding algorithms using neural networks since they provide low dimension representation of networks using paths, as the proposed method (Khosla et al., 2019; Narayanan et al., 2017).

But, beyond technical concerns, ‘TL‐tsne’ layout algorithm is suitable for trophic networks since it allows reading and interpreting the network along fixed axes contrary to traditional force‐based layouts. These axes have an ecological interpretation involving energy diffusion in the network. More precisely, the first axis, the trophic levels, describes the hierarchy in the acquisition of resources. Although this scalar quantity is not enough to summarise the network as pointed by the criticisms of this concept (Cousins, 1987), it is in line with a thermodynamic interpretation of trophic networks (Lindeman, 1942; Thompson et al., 2012). Using trophic level as first axis to represent trophic networks is almost consensual, as in the function PlotWebByLevel() from the ‘cheddar’ R package (Hudson et al., 2013) or in Potapov (2022). Contrary to ‘cheddar’ where the second axis is implicit or to Potapov (2022) where it is a species trait, the second axis in our layout represents an explicit complementary information related to diffusion of energy along the network and that can be computed without additional information about species. In our ‘TL‐tsne’ layout, two species with similar trophic level may have different *y*‐axis values, which indicates that they belong to different energetic channels. Such a pattern is illustrated in the Angola coastal network and Norway soil network where the ‘TL‐tsne’ layout highlights two distinct channels for both networks: the green channel, linked to primary producers, (either phytoplankton or plants) and the brown channel, linked to detritus (Moore et al., 2004; Mougi, 2020; Polis & Strong, 1996). To our knowledge, this is the first network layout algorithm that highlights these channels on empirical trophic network data. This sheds new lights on a common structure shared by coastal and terrestrial communities, as previously suggested in the literature (Bramon Mora et al., 2018). Moreover, the diffusion parameter *β*, allows accentuating the separation between these different channels, as shown in Figure 2. Although the parameter *β* can be optimised numerically using extended Moran index, we however encourage the user of *metanetwork* to explore several *β* configurations in order to represent channels gradually separated from each other.

As a conclusion, our layout method based on diffusion processes, which highlights ecological processes such as organic matter diffusion, emphasises meaningful structures for trophic ecology. We insist on the fact that network representation goes beyond visualisation (e.g. Pawluczuk & Iskrzyński, 2022) because it also deals with network layout problem. In addition, our package allows dealing with different scales of the metanetwork. This may help for instance for the understanding of the effect of environmental changes at different spatial scales or different aggregation levels. On top of that, we have developed operations on the network, which allow comparing networks at different location or different time. Thus, the present package, thanks to network representation, manipulation and comparison tools should help practitioners to better explore trophic metanetworks.

## AUTHOR CONTRIBUTIONS

**Marc Ohlmann:** Conceptualization (equal); methodology (equal); software (lead); visualization (lead); writing – original draft (lead). **Jimmy Garnier:** Conceptualization (equal); methodology (equal); software (supporting); supervision (equal); visualization (supporting); writing – original draft (supporting). **Laurent Vuillon:** Conceptualization (equal); methodology (equal); software (supporting); supervision (equal); visualization (supporting); writing – original draft (supporting).

## CONFLICT OF INTEREST STATEMENT

The authors declare no conflict of interest.

## Supporting information


Appendix S1

## Data Availability

This paper uses a simulated data set available as a vignette of the package documentation available online (https://marcohlmann.github.io/metanetwork/articles/pyramid.html). It also uses three data sets that are already available in the package: Angola coastal network: data set is extracted from Web of Life (https://www.web‐of‐life.es/map.php?type=7), is attached to *metanetwork* and analysed in a vignette (https://marcohlmann.github.io/metanetwork/articles/angola.html). Norway soil network: this data set from is attached to *metanetwork* and analysed in a vignette (https://marcohlmann.github.io/metanetwork/articles/norway.html). European vertebrate metaweb: this data set from is attached to *metanetwork* and analysed in a vignette (https://marcohlmann.github.io/metanetwork/articles/vertebrates.html). All the analysis of the datasets using the R package metanetwork are available online: Angola coastal network analysis https://github.com/MarcOhlmann/metanetwork/blob/HEAD/vignettes/angola.Rmd, Noway soil network analysis https://github.com/MarcOhlmann/metanetwork/blob/HEAD/vignettes/norway.Rmd, European Vertebrate metaweb analysis https://github.com/MarcOhlmann/metanetwork/blob/HEAD/vignettes/vertebrates.Rmd.
