# Supplementary material for: metanetwork: A R package dedicated to handling and representing trophic metanetworks
Source: Ecol Evol. 2023 Aug 15;13(8):e10229. doi: 10.1002/ece3.10229 (PMC10427773; doi:10.1002/ece3.10229)
Supplement: Supplementary file 1 — Appendix S1 [file ECE3-13-e10229-s001.pdf]

Supporting Information: *metanetwork*, a R package dedicated to handling and representing trophic metanetworks

Marc Ohlmann, Jimmy Garnier, Laurent Vuillon

1 Computing trophic levels

MacKay *et al.* (2020) have defined trophic level using Laplacian matrix of trophic networks. We detail here the definition, the properties and the computation of trophic levels on disconnected local networks in our package 'metanetwork'.

Let $G^\star$ be the directed metaweb of the considered metanetwork that is assumed to be weakly connected. We note $\mathbf{A}$ its adjacency matrix and $\mathbf{D}$ its total degree diagonal matrix. The laplacian matrix of the undirected version of $G^\star$ is defined by:

$$\mathbf{L} = \mathbf{D} - \mathbf{A} - t(\mathbf{A}) \tag{1}$$

where $t(\mathbf{A})$ is the transpose of the matrix $\mathbf{A}$. The number of connected components of $G^\star$ is equal to the multiplicity of the eigenvalue $\mathbf{0}$ for $\mathbf{L}$ (see Marsden 2013). Since the metaweb is connected, the eigenvalue 0 is simple, that is:

$$\dim(\ker \mathbf{L}) = 1 \tag{2}$$

Moreover, since the rows and columns of $\mathbf{L}$ sum to 0, we deduce that $\mathbf{e} = (1, \ldots, 1)$ is the associated eigenvector, $\mathbf{Le} = \mathbf{0}$.

Now, we introduce the imbalance vector $\mathbf{v}$ defined as follows

$$\mathbf{v} = \text{indegree}(G) - \text{outdegree}(G)$$

where $\text{indegree}(G)_i$ is the number of vertices ending in the nodes i and $\text{outdegree}(G)$ is the number of vertices starting from the nodes i. The vector $\mathbf{v}$ captures the difference between the number of species or groups, which depends on you and the number of species or groups, on which you rely on. MacKay *et al.* (2020) defines the *trophic levels* $\mathbf{x}$ of a network G as the solution of the following linear system:

$$\mathbf{Lx} = \mathbf{v}. \tag{3}$$

Since the dimension of $\ker(\mathbf{L})$ is 1, the solution $\mathbf{x}$ is unique up to an additive constant. As a result, we see that trophic levels are defined relatively to a given species and it is not absolute.

In our 'metanetwork' package, we fix the constant such that the the lowest trophic level is equal to 0, that is we solve the system $\mathbf{L}\mathbf{x} = \mathbf{v}$ under the constraint that $\min(\mathbf{x}) = 0$.

Local networks can be disconnected due for instance to sampling effects or diversity loss. In that case, the dimension set of the solutions to the system (1) is equal to the number of connected components of the local network. Thus in each connected component, the solution of the induced problem is unique up to an additive constant that is different for each connected component. In our package 'metanetwork', we use the connected graph $G^\star$, which contains all the local networks to fix those constants. More precisely, in $G^\star$ we have fixed the trophic level of any node. Thus, to fix the constant in each connected component of the local network, we set the lowest trophic level in this component to its value in the metaweb $G^\star$. In the function `compute_TL`, we compute trophic levels recursively on the connected components of the local networks.

2 'TL-tsne' algorithm

In this section, we provide more details on the implementation of our TL-tsne layout algorithm, which is inspired from the t-sne algorithm. The main idea is to use a reduction dimension method based on the minimisation of Kulbach-Leilber distance between a high dimensional similarity matrix and a low dimensional similarity matrix associated to the focusing network G.

We use the diffusion graph kernel (Kondor & Lafferty 2002), $\mathbf{K}$, associated to the network G as a similarity matrix in high dimension. It is defined as:

$$\mathbf{K} = \exp(-\beta \mathbf{L}) = \sum_{k \geq 0} \frac{(-\beta \mathbf{L})^k}{k!} \tag{4}$$

where $\mathbf{L}$ is the Laplacian matrix of G and β is the diffusion parameter, a scalar and strictly positive parameter. The dimension of $\mathbf{K}$ is $n \times n$ where n is the number of nodes in the network G.

In the low dimensional space, pairwise similarity between two nodes i and j is measured as in the 't-sne' methods. In the low dimensional space of dimension 2, each node is characterized by a vector $\mathbf{z}_i = (x_i, y_i)$, where x_i is the trophic level of the node, which is already fixed, and y_i is the y-axis coordinate which is unknown. In this low dimensional space, the similarity matrix

$\mathbf{Q} = (q_{i,j})$ is defined for all $i, j \in \{1, \ldots, n\}$ by:

$$q_{i,j} = \frac{(1 + \|\mathbf{z}_i - \mathbf{z}_j\|^2)^{-1}}{\sum_{k \neq i}(1 + \|\mathbf{z}_i - \mathbf{z}_k\|^2)^{-1}}, \quad \text{for all} \quad j \neq i \quad \text{and} \quad q_{i,i} = 0. \tag{5}$$

The cost function to optimise consists in the Kullbach-Leibler divergence between diffusion graph kernel $\mathbf{K}$, which is the similarity matrix in high dimensional space, and the similarity in low dimensional space $\mathbf{Q}$. This function $C(\mathbf{y})$ depends on the unknown y-axis coordinates $\mathbf{y} = (y_i)$ and it is defined by

$$C(\mathbf{y}) = KL(\mathbf{K}\|\mathbf{Q}(\mathbf{y})) = \sum_i \sum_{j \neq i} K_{i,j} \log\left(\frac{K_{i,j}}{q_{i,j}(\mathbf{y})}\right) = \sum_i \sum_{j \neq i} K_{i,j} \log(K_{i,j}) - \sum_i \sum_{j \neq i} K_{i,j} \log(q_{i,j}(\mathbf{y})) \tag{6}$$

We can now compute the gradient $\frac{\delta C}{\delta y_i}$ of the cost function with respect to any unknown coordinate vector $\mathbf{y}$ (see Van der Maaten & Hinton 2008 for details),

$$\frac{\delta C}{\delta y_i}(\mathbf{y}) = 4 \sum_{j \neq i} \big(K_{i,j} - q_{i,j}(\mathbf{y})\big)(y_i - y_j)(1 + \|\mathbf{z}_i - \mathbf{z}_j\|^2)^{-1}. \tag{7}$$

We can observe that the line $\mathbb{R}\mathbf{e}$ with $\mathbf{e} = (1, \ldots, 1)$ is always an extremum of C. Moreover, if $\mathbf{K}$ is the matrix with all coefficients equal to 1, the only minimum of C up to a multiplicative constant is $\mathbf{e}$. Since $\mathbf{K}(\beta)$ converges toward this matrix with all coefficient equal to 1 as $\beta \to \infty$ (it results from the Perron-Frobenus theorem and properties of the Laplacian matrix L), the vector $\mathbf{y}$ of the TL-tsne algorithm converges to $\mathbf{e}$ when $\beta \to \infty$. We now provide the 'TL-tnse' layout algorithm in pseudo code,

Algorithm 1: TL-tsne layout algorithm for trophic networks

Input: G (directed network, n nodes), $\beta \in (0, \infty)$

Optimisation parameters: number of iterations T, learning rate η, momentum $\alpha(t)$

Output: node embedding $\mathbf{z}^{(T)} = (\mathbf{x}, \mathbf{y}^{(T)})$ (dimension $n \times 2$)

Compute trophic levels $\mathbf{x}$ by solving $\mathbf{Lx} = \mathbf{v}$;

Compute diffusion graph kernel $\mathbf{K} = \exp(-\beta\mathbf{L})$;

Initialisation: set $\mathbf{z}^{(0)}[, 1] = \mathbf{x}$;

Sample initial solution $\mathbf{y}^{(0)}$ from $\mathcal{N}(\mathbf{0}, 10^{-4}\mathbf{I}_n)$ and set $\mathbf{z}^{(0)}[, 2] = \mathbf{y}^{(0)}$;

for $t = 1$ *to* T **do**

  Compute low dimensional affinities $q_{i,j}$ using Eq. 5 ;

  Compute the gradient ∇C of the cost function (single axis) using Eq. 7;

  Set $\mathbf{y}^{(t)} = \mathbf{y}^{(t-1)} + \eta\nabla C + \alpha(t)(\mathbf{y}^{(t-1)} - \mathbf{y}^{(t-2)})$

end

3 Selecting diffusion parameter β using Moran index

In this section, we introduce a quantitative method to select an optimal value for the parameter β. Intuitively, on the pyramid example, increasing the value of the diffusion parameter β tends to bring together, on the y-axis, nodes belonging to the same channel ('A,B,C', 'D,E,F,G,H,I,J' and 'K,L,M,N,O') and to move away these groups of nodes from each other.

Originally introduced to measure spatial autocorrelation (De Jong *et al.* 1984), Moran index helps us in assessing the quality of the computed layout in function of β parameter and thus choose an optimal β value. More precisely, given a network G and a parameter β, we can compute the y-axis vector $\mathbf{y}(\beta)$ using the 'TL-tsne' algorithm. Then we can define the Moran Index $\mathrm{I}(\beta)$ of the vector $\mathbf{y}(\beta)$ associated to the adjacency matrix $\mathbf{A}$ of the network G as follows

$$\mathrm{I} = \frac{n}{\sum\limits_{q,l} A_{ql}} \frac{\sum_{q,l} A_{ql}(y_q - \overline{y})(y_l - \overline{y})}{\sum_q (y_q - \overline{y})} \tag{8}$$

where $\overline{y}$ denotes the mean of $\mathbf{y}$. The Moran index I varies between -1 and 1 and it measures the y-axis correlation between the neighbors of the network G. Since increasing β squeezes the

layout and so increases correlation of the y-axis while making visualisation unreadable (nodes are then overlapping ~~see Supporting Information~~), we introduce an extended Moran index I_e which penalises node overlap. Denoting $\epsilon > 0$ the label size of the current network and $\mathbf{D} = (\|y_q - y_l\|)_{q,l}$ the Euclidian distance matrix built from $\mathbf{y}$, we define:

$$\mathrm{I}_e = \frac{n}{\sum_{q,l} A_{ql}} \frac{\sum_{q,l} A_{ql}(y_q - \overline{y})(y_l - \overline{y})}{\sum_q (y_q - \overline{y})} - \frac{1}{n^2} \sum_{q,l} \mathbf{1}_{\{\|y_q - y_l\| \leq \epsilon\}} \tag{9}$$

where $\mathbf{1}_{\{\}}$ denotes the indicator function. This index becomes close to 0 when β is either very large or very low compare to the dominant eigenvalue of the network. Thus it reaches a maximum for a positive value of β.

We computed the extended Moran index for the pyramid network example (see Fig. S1). We used 100 β values between 0.001 and 0.5 and we averaged the extended Moran index over 20 repetitions (since the 'TL-tsne' layout algorithm is stochastic). For low β values, the outcome of the 'TL-tsne' layout algorithm varies a lot but, on average, the neighbors do not have correlated y-axis values, leading to low I_e values and high standard deviation (Fig. S1a, Fig. S2). Similarly, for high β values, the neighbors do have correlated y-axis values but the nodes are overlapping, penalizing the extended Moran index, which becomes very low (see Fig.S1c). However, for intermediate β values (around 0.1 for the example of Fig. S1b), neighbors have correlated y-axis values, which highlights the hierarchical structure of the network, but the nodes does not overlap, which leads to high index I_e values.

We followed the same procedure to assess the quality of the 'TL-tsne' layout (Fig. S3, Fig. S4). We used 100 β values between 0.001 and 0.5 and we averaged the extended Moran index over 20 repetitions

References

De Jong, P., Sprenger, C. & Van Veen, F. (1984) On extreme values of moran's i and geary's c. *Geographical Analysis*, **16**, 17–24.

Kondor, R.I. & Lafferty, J. (2002) Diffusion kernels on graphs and other discrete structures.

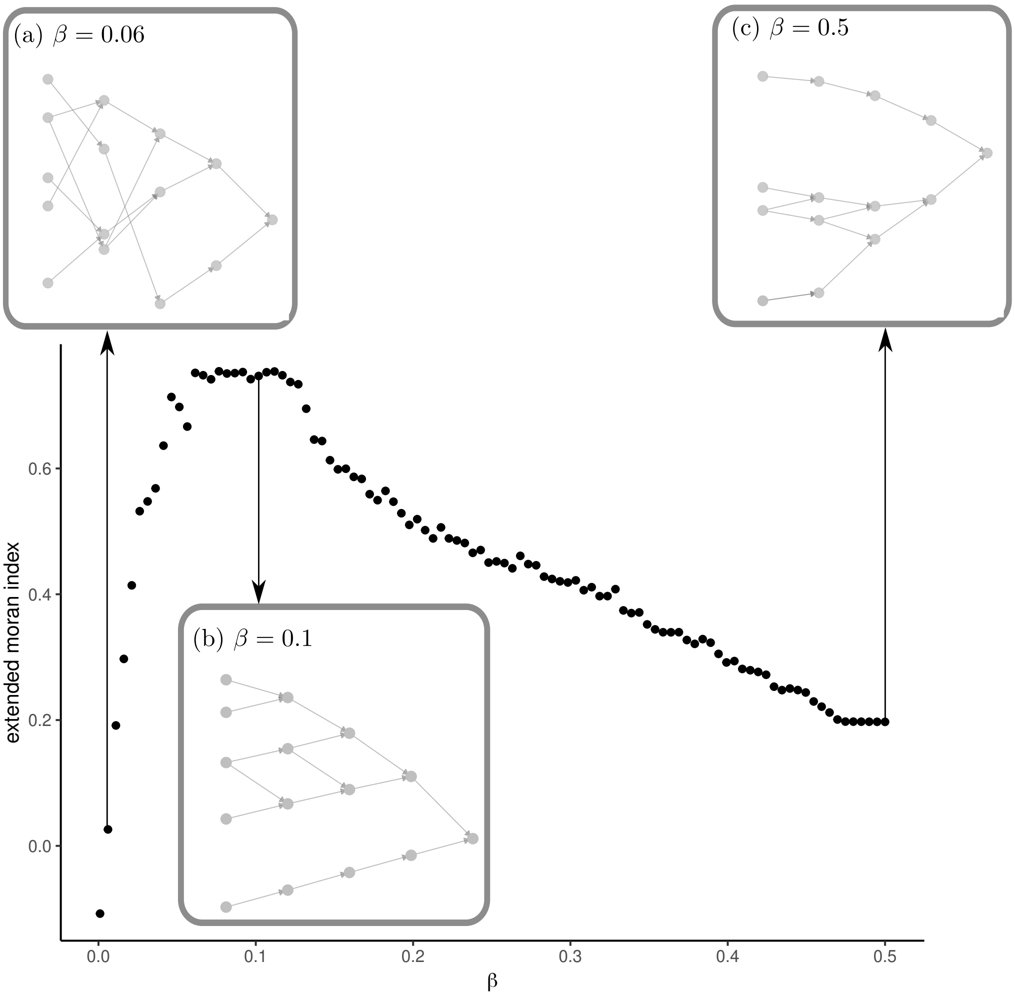

Figure S1: Extended Moran index as a function of the diffusion parameter β for the pyramid network example presented in Fig. 2. In panel (a), low β values ($\beta = 0.06$) lead to unstable representations (*i.e.* neighbors do not have correlated positions). In panel (b), intermediate β values ($\beta = 0.1$) favor stable and meaningful representations. In panel (c), high β values ($\beta = 0.5$) lead to stable but crowded representations (*i.e.* with clustered and overlapping nodes)

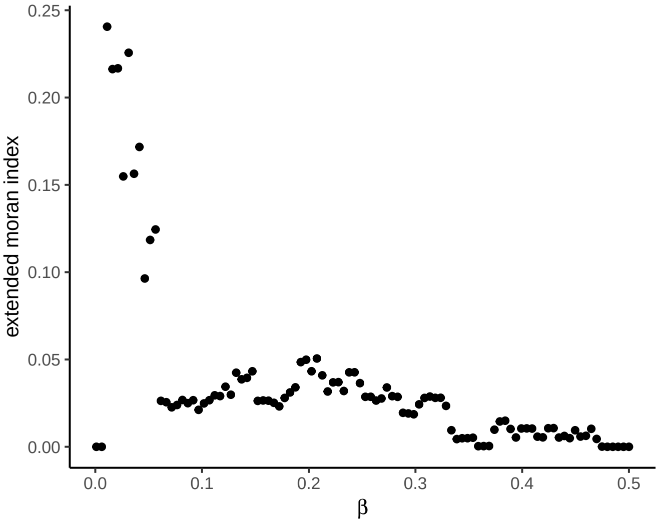

Figure S2: Standard deviation of the extended Moran index as a function of the diffusion parameter β for the pyramid example

Proceedings of the 19th international conference on machine learning, volume 2002, pp. 315–322.

MacKay, R.S., Johnson, S. & Sansom, B. (2020) How directed is a directed network? *Royal Society open science*, **7**, 201138.

Marsden, A. (2013) Eigenvalues of the laplacian and their relationship to the connectedness of a graph. *University of Chicago, REU.*

Van der Maaten, L. & Hinton, G. (2008) Visualizing data using t-sne. *Journal of machine learning research*, **9**.

4 Supplementary figures

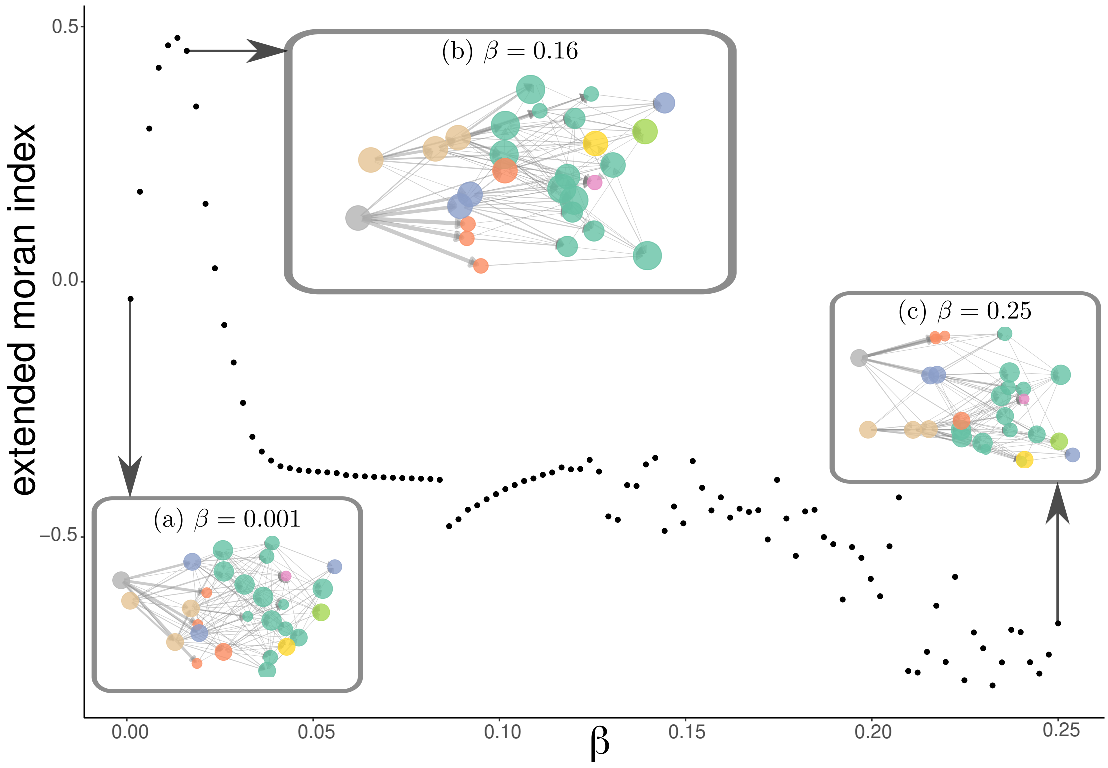

Figure S3: Extended Moran index as a function of the diffusion parameter β for the Angola data set. In panel (a), low β values ($\beta = 0.001$) lead to unstable representations (*i.e.* neighbors do not have correlated positions). In panel (b), intermediate β values ($\beta = 0.16$) favor stable and meaningful representations. In panel (c), high β values ($\beta = 0.25$) lead to stable but crowded representations (*i.e.* with clustered and overlapping nodes)

Species	Group	File	Ref_phylopic	Credit
Calonectris diomedea	1	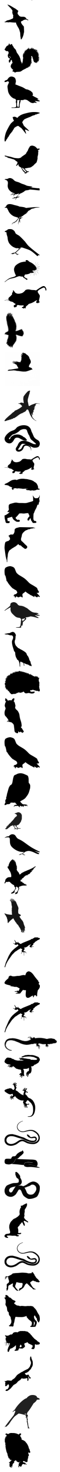	http://phylopic.org/image/a8ecc1e9-f339-4d85-84c3-aa2fff47b2c1/	Juan Carlos Jerí
Scurius vulgaris	2		http://phylopic.org/image/2916f8fe-290a-423b-b6f7-fb975c30d518/	Andy Wilson
Larus argentatus	3		http://phylopic.org/image/966db6c3-7719-400d-af61-a2d671b293b8/	Rebecca Groom
Apus apus	4		http://phylopic.org/image/3e6b9118-7aff-4b64-8c95-38b2ee49d922/	Ferran Sayol
Acrocephalus	5		http://phylopic.org/image/67a9ecfd-58ba-44a4-9986-243b6e610419/	uncredited
Monticola saxatilis	6		http://phylopic.org/image/14962f0a-c7db-4df2-89e3-6cfc593faed1/	Francesco Architetto Rollandin
Prunella collaris	7		http://phylopic.org/image/88aeb901-6979-40a9-8991-b33dd8ac0a38/	Matt Wilkins
Emberiza cirlus	8		http://phylopic.org/image/42fdc3cb-37fc-4340-bdf9-eed8e050137c/	L. Shyamal
Microtus arvalis	9		http://phylopic.org/image/f7d6d04c-73fa-4bf3-8c94-48134e6857b9/	uncredited
Mus spretus	10		http://phylopic.org/image/6b2b98f6-f879-445f-9ac2-2c2563157025/	Madeleine Price Ball
Buteo buteo	11		http://phylopic.org/image/5c90ad4f-f3fc-4210-ad27-e8ea01f5c2f6/	Andy Wilson
Accipiter nisus	12		http://phylopic.org/image/92589388-08e3-422f-b452-aa7454411a9c/	Beth Reinke
Chlidonias niger	13		http://phylopic.org/image/06ae006e-1ad5-4f3d-ab40-f6d91ea24a7f/	terngirl
Natrix natrix	14		http://phylopic.org/image/418e6939-dc41-4424-a646-5ba1fb276648/	V. Deepak
Meriones crassus	15		http://phylopic.org/image/6b2b98f6-f879-445f-9ac2-2c2563157025/	Madeleine Price Ball
Talpa caeca	16		http://phylopic.org/image/0465d81c-0def-4478-af15-a075d472e957/	Steven Traver
Lynx lynx	17		http://phylopic.org/image/27a2173a-5903-46fc-83c5-29ed7f421046/	Margot Michaud
Falco columbarius	18		http://phylopic.org/image/6cebf754-cb71-448d-a5bb-947157205264/	Liftarn
Nyctea scandiaca	19		http://phylopic.org/image/ea71ef37-b1ce-428b-84d0-438d0d954f32/	xgirouxb
Haematopus ostralegus	20		http://phylopic.org/image/65ed3e2d-38f5-421f-976c-be4df6ac73fa/	terngirl
Ardea cinerea	21		http://phylopic.org/image/c91c655d-a2d6-485d-8702-b3143e1eaf81/	Nina Skinner
Erinaceus europaeus	22		http://phylopic.org/image/f83c6893-f0ed-4ec4-b558-aa774c5c9b5b/	Claus Rebler
Asio otus	23		http://phylopic.org/image/2079bb77-eadb-49dc-950e-96a0514dfa4c/	Ferran Sayol
Strix aluco	24		http://phylopic.org/image/ea71ef37-b1ce-428b-84d0-438d0d954f32/	xgirouxb
Surnia ulula	25		http://phylopic.org/image/4e35207e-a75a-4518-8eca-1d574eebfbb4/	Ferran Sayol
Corvus monedula	26		http://phylopic.org/image/d1f1314f-51bf-433c-80c8-e83208ce856a/	Ferran Sayol
Pyrrhocorax pyrrhocorax	27		http://phylopic.org/image/34561529-1ff5-4c20-96ce-ce946e86cbfa/	L. Shyamal
Stercorarius skua	28		http://phylopic.org/image/e57d083f-9885-4900-90ba-e1e9cf61230a/	Liam Quinn and Albertonykus
Milvus milvus	29		http://phylopic.org/image/63f337b6-8a58-4939-ba8b-f1bd3b8c1037/	uncredited
Podarcis lilfordi	30		http://phylopic.org/image/d4c856c6-49b3-4b48-ad1f-1bbbc8cf58fa/	uncredited
Discoglossus montalentii	31		http://phylopic.org/image/081b3539-a72e-40b3-9102-0e546fadf545/	Nobu Tamura and T. Michael Keesey
Podarcis bocagei	32		http://phylopic.org/image/d4c856c6-49b3-4b48-ad1f-1bbbc8cf58fa/	uncredited
Lacerta viridis	33		http://phylopic.org/image/18af6753-2f5b-49d2-a28d-4cc1b7deaf6f/	Gustav Mützel
Phrynocephalus guttatus	34		http://phylopic.org/image/e246fc36-cb24-4a87-8292-41f55b9dcc0e/	Beth Reinke
Tarentola bischoffi	35		http://phylopic.org/image/264fa655-afd7-451c-8f27-e0a9557376e6/	Birgit Szabo
Hierophis gemonensis	36		http://phylopic.org/image/d38e6764-b49c-4728-aacd-2f8bbd26718e/	Ignazio Avella
Vipera aspis	37		http://phylopic.org/name/2a876f61-1847-40e8-a0fe-77258d3cca18/	Alex Slavenko
Vipera berus	38		http://phylopic.org/image/a1cd1814-e0b4-409c-af18-8cb2f841569c/	Beth Reinke
Mustela eversmanii	39		http://phylopic.org/image/a67f24a9-6d75-420c-b437-6817da5cec1a/	uncredited
Dolichophis schmidti	40		http://phylopic.org/image/d38e6764-b49c-4728-aacd-2f8bbd26718e/	Ignazio Avella
Sus scrofa	41		http://phylopic.org/image/87047da1-b40e-4b31-8492-4db262f129f5/	Ferran Sayol
Canis lupus	42		http://phylopic.org/image/e4e306cd-73b6-4ca3-a08c-753a856f7f12/	Tracy A. Heath
Felis silvestris	43		http://phylopic.org/image/1edbe9ff-c453-47f3-9174-2e1f9c5983de/	Steven Traver
Genetta genetta	44		http://phylopic.org/image/8884990f-2295-448f-ada8-81625ef6dead/	Margot Michaud
Lanius excubitor	45		http://phylopic.org/image/4d96aa6b-ef47-48c7-a069-ac01668fbf67/	uncredited
Bubo bubo	46		http://phylopic.org/image/debc4910-7742-42ac-a782-4c449fd1e882/	Lukasiniho

Table S1: Species chosen to represent each Stochastic Block Model group for the legend of Fig. 6 (main text), its silhouette taken from phylopic (http://phylopic.org) and the corresponding credit

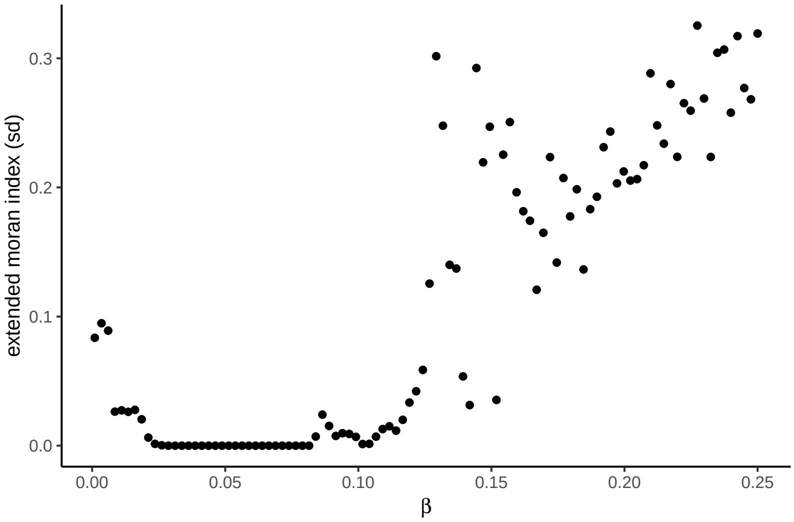

Figure S4: Standard deviation of the extended Moran index as a function of the diffusion parameter β for the Angola data set.

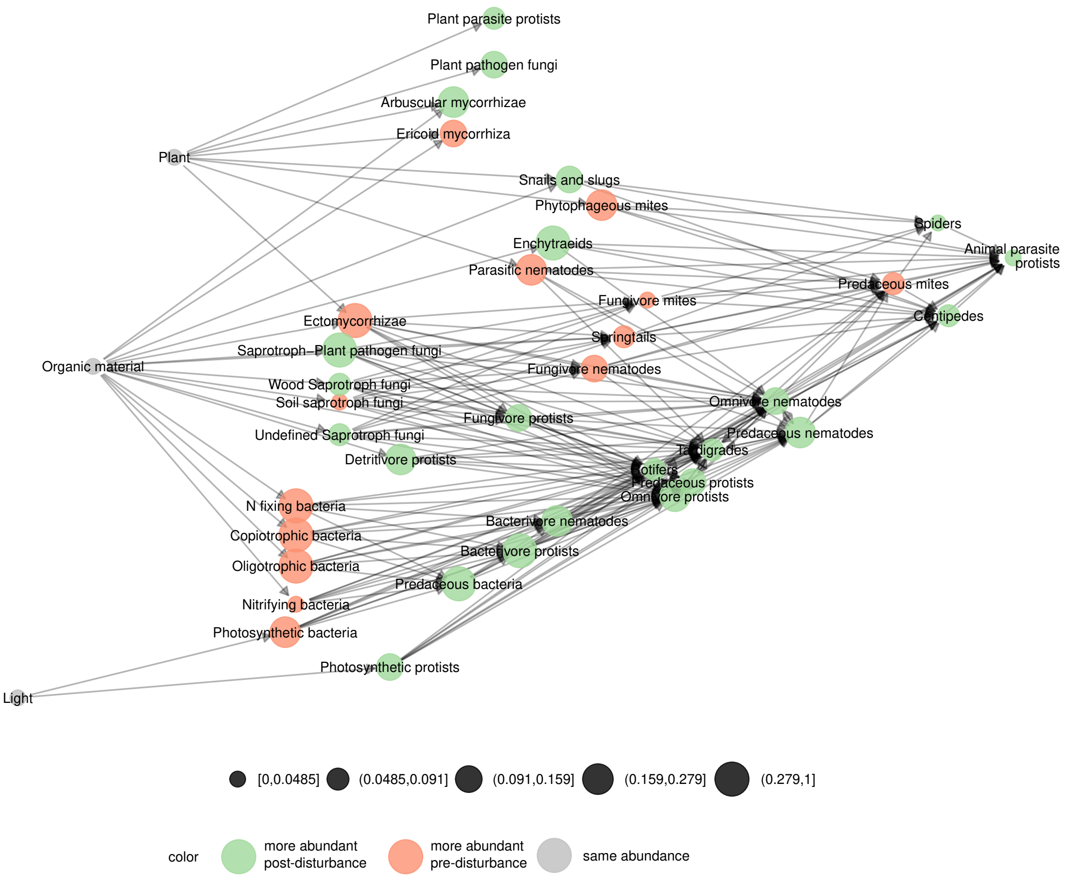

Figure S5: Difference network between Norway soil network in disturbed and non-disturbed sites. Differences in node abundances are built from eDNA data. We use the `diff_plot` function with the computed 'TL-tsne' metaweb layout (see `layout_metaweb` option) to visualize the difference network.

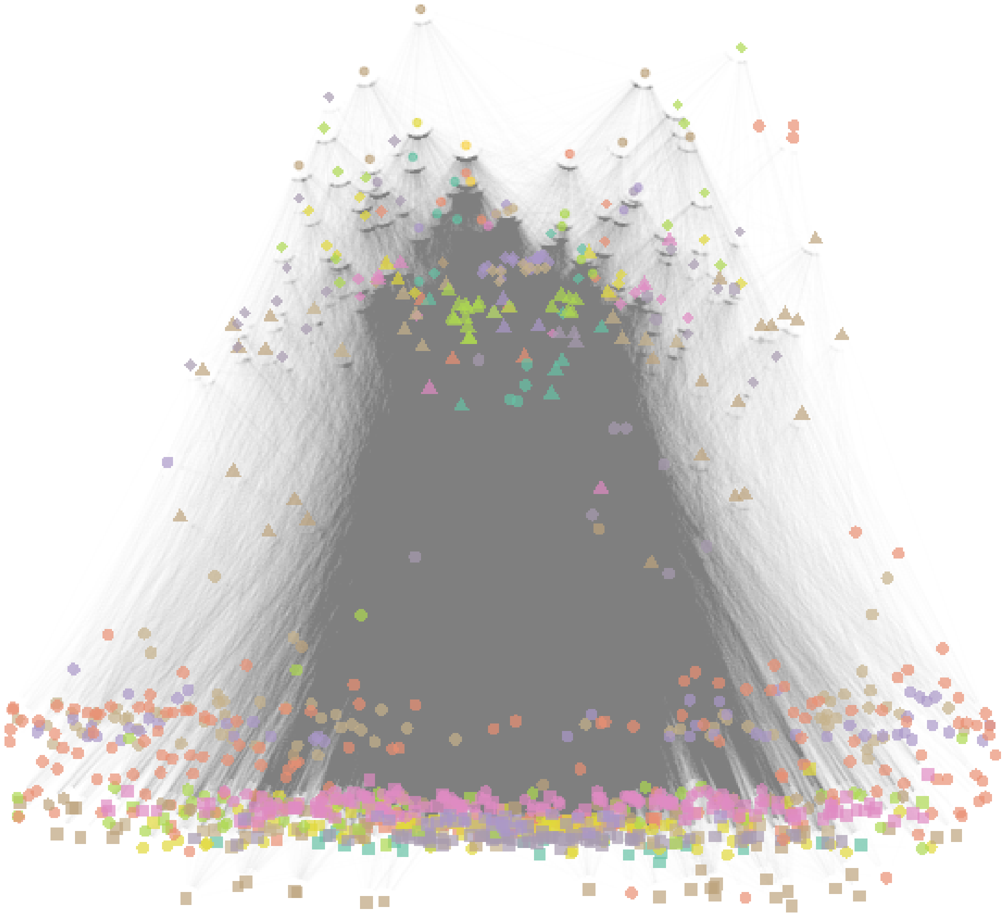

Figure S6: Metaweb of European tetrapods, with 1101 species (mammals, breeding birds, reptiles and amphibians) and 48963 interactions, with 'TL-tsne' layout ($\beta = 0.005$)

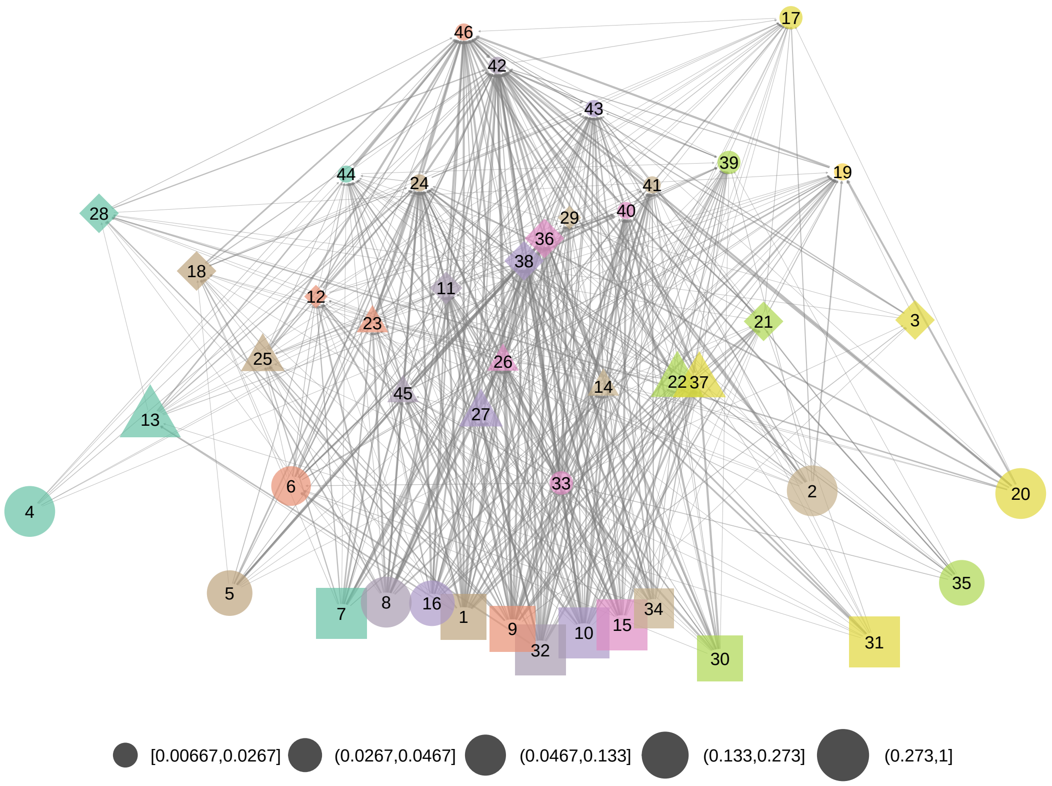

Figure S7: Metaweb of European tetrapods at a Stochastic Block Model level (46 groups). Nodes have colors and shapes corresponding to estimated Stochastic Block Model groups. It is represented using 'TL-tsne' layout ($\beta = 0.005$) and `ggmetanet` visualisation. In this representation, the y-axis is the trophic level.
